# Correction loss following short-segment posterior fixation for traumatic thoracolumbar burst fractures related to endplate and intervertebral disc destruction

**DOI:** 10.1186/s12891-023-06288-y

**Published:** 2023-03-08

**Authors:** Takumi Hashimura, Eijiro Onishi, Satoshi Ota, Yoshihiro Tsukamoto, Shinnosuke Yamashita, Tadashi Yasuda

**Affiliations:** Department of Orthopedic Surgery, Kobe City Medical Center General Hospital 2-1-1 Minamimachi, Minatojima, Chuo-ku, Kobe City, Hyogo prefecture 650-0047 Japan

**Keywords:** Thoracolumbar burst fractures, Short-segment posterior fusion, Correction loss, Traumatic intervertebral disk lesion, Endplate injury

## Abstract

**Background:**

There has been widespread use of short-segment posterior fixation (SSPF) for traumatic thoracolumbar burst fractures. The relationship between the destruction of the vertebral endplate and adjacent disc and postoperative correction loss has been studied in only a few studies. This study investigated the risk factors for correction loss following SSPF.

**Methods:**

Forty-eight patients (mean age 35.0 years) who underwent SSPF for thoracolumbar burst fractures were enrolled. The mean follow-up period was 25.7 months (12–98 months). The neurological status and postoperative back pain were assessed by the medical records. Segmental kyphotic angle (SKA) and anterior vertebral body height ratio (AVBHR) were measured radiographically to assess indirect vertebral body reduction and local kyphosis. Preoperative Sander’s traumatic intervertebral disc lesion (TIDL) classification and AO classification were used to evaluate the severity of disc and vertebral endplate injury. The corrective loss was considered present if ΔSKA was ≥10°. A multivariate logistic regression analysis was performed to identify the risk factors associated with postoperative loss of correction.

**Results:**

The fracture distribution was as follows: 10 at T12, 17 at L1, 10 at L2, 9 at L3, and 2 at L4. Vertebral fractures were classified in the following way: A3 in 13 patients, A4 in 11, B1 in 11, and B2 in 13. In 47 patients (98%), a union of the fractured vertebrae was achieved. SKA and AVBHR improved significantly after surgery from 11.6° to 3.5° and from 67.2 to 90.0%, respectively. However, the correction loss at follow-up was 10.4° and 9.7%, respectively. Twenty patients (42%) had severe TIDL (grade 3). Postoperative ΔSKA and ΔAVBHR were significantly higher in patients with TIDL grade 3 than with TIDL grade 0–2. The presence of cranial TIDL grade 3 and older age were significant risk factors for ΔSKA ≥10° on multivariate logistic regression analysis. All patients could walk at follow-up. TIDL grade 3 and ΔSKA ≥10° were associated with severe postoperative back pain.

**Conclusions:**

Risk factors for loss of correction after SSPF for thoracolumbar burst fractures were severe disc and endplate destruction at the time of injury and older age.

## Background

Thoracolumbar burst fractures are common spinal injuries usually caused by high-energy trauma, sometimes accompanied by neurological complications. Although various surgical procedures have been used to treat these spinal injuries, definitive treatment methods remain controversial [1–3]. Nevertheless, surgical treatment aims to improve neurological deficits, reduce the deformed spine, and prevent future kyphotic deformity.

Short-segment posterior fixation (SSPF) for traumatic thoracolumbar burst fractures has been widely used because of its minimally invasive technique and efficacy in reducing kyphotic deformity while preserving the motion segment [1, 2], however, postoperative correction loss remains a concern. Risk factors for correction loss after SSPF have been discussed [4–8]. Previous studies have demonstrated that most correction loss occurs at the intervertebral disc level and not in the vertebral body [4, 9], which may be supported by the report that endplate injuries are the leading cause of degeneration in damaged discs [10]. Therefore, assessing intervertebral disc and adjacent endplate damage is crucial for determining viable treatment methods and predicting delayed kyphotic deformity. If correction loss after surgery is highly suspected, anterior reconstruction surgery or long-segment posterior fixation (LSPF) could be preferred procedures. The assessment of traumatic intervertebral disk lesions (TIDLs) based on magnetic resonance imaging (MRI) has been proposed to evaluate damaged intervertebral discs [9–13]. However, few studies have addressed the association between intervertebral disc injury and postoperative delayed kyphotic deformity due to correction loss.

This study investigated risk factors for correction loss following SSPF for thoracolumbar burst fracture, especially concerning endplate damage of the fractured vertebrae and adjacent discs.

## Materials and methods

### Patient demographics

This retrospective study using anonymized data with a general opt-out procedure was approved by the institutional review board (Approval number: zn210817). All procedures performed in this study were in accordance with the ethical standards of our institutional ethics committee, and with the 1964 Helsinki Declaration and later amendments or comparable ethical standards. The need for informed consent was waived because of the retrospective study design. Patients who underwent SSPF for traumatic thoracolumbar burst fracture between October 2011 and August 2020 were included. The exclusion criteria were as follows: osteoporotic fractures, multiple vertebral burst fractures, patients with less than 12 months of follow-up, and those treated with anterior reconstruction or LSPF. Ultimately, 48 patients were enrolled in this study.

### Surgical technique

All surgical procedures were performed under general anesthesia. Patients were placed in the prone position. Using the Wiltse paraspinal approach [14], pedicle screws (CD Horizon Solera Sagittal Adjusting Screw®, Medtronic) were placed in the vertebrae below and above the fracture. After attaching the rod, indirect reduction via ligamentotaxis was performed to restore the vertebral body height and achieve posterior wall decompression. Postero-lateral fusion or facet fusion was not applied. After reduction maneuver, seventeen patients who showed intra-vertebral cavities underwent vertebroplasty by filling the hydroxyapatite blocks through the trans-pedicular approach. Seven patients who showed severe canal compromise by the posterior wall fragment before surgery underwent additional posterior decompression. Anterior decompression through a posterior approach was not performed.

### Assessment of clinical outcomes

Neurological conditions were assessed using the American Spine Injury Association (ASIA) impairment scale [15]. In addition, postoperative low back pain was classified according to the Japanese Orthopaedic Association (JOA) scoring system as follows: no pain (3 points), occasional minor back pain (2 points), constant back pain or sometimes severe back pain (1 point), and constant severe back pain (0 points) [16]. In this study, postoperative severe back pain was defined as positive in patients with zero or one point.

### Radiographic assessment

Plain radiographs and computed tomography (CT) scans were obtained before surgery, immediately after surgery, at the removal of the implants, and during the final follow-up. The segmental kyphotic angle (SKA) and anterior vertebral body height ratio (AVBHR) were measured as radiographic parameters to evaluate the indirect reduction of the vertebral body and local kyphosis. SKA was defined as the Cobb angle calculated between the cranial vertebra’s upper endplate and the caudal vertebra’s lower endplate. AVBHR was defined as the percentage of the anterior vertebral height of the fractured vertebra to the average anterior height of the two adjacent vertebrae (Fig. 1) [17].

**Fig. 1:**
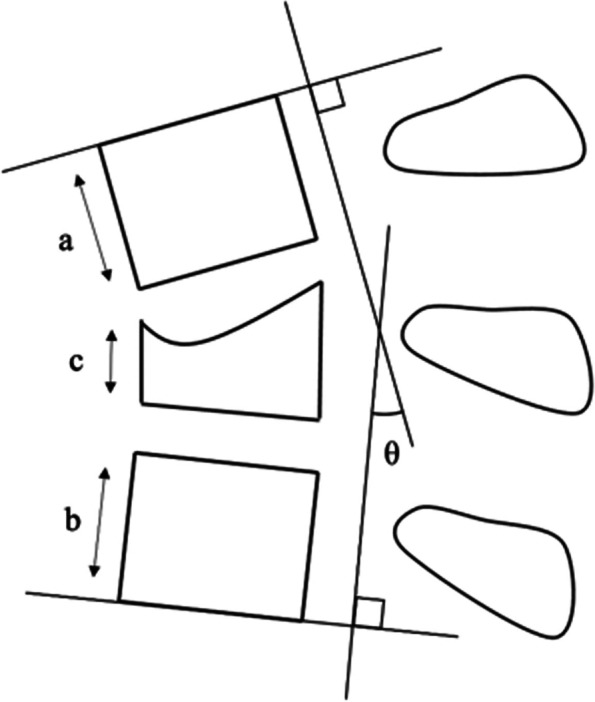
Schematic diagrams of radiographic parameters. Segmental kyphotic angle (SKA) = θ, Anterior vertebral body height ratio (AVBHR) = c/(a + b)/2

The indirect reduction of fractured vertebrae and correction loss during observation were evaluated using SKA and AVBHR. In this study, correction loss was considered present if the ΔSKA was ≥10° immediately after surgery to the final examination [4, 6].

We evaluated the degree of vertebral body involvement using the load sharing classification (LSC) scoring system [18]. The vertebral fractures were classified according to the AO classification system [19]. The severity of intervertebral disc and vertebral endplate injury were assessed using the preoperative Sander’s TIDL classification based on T2-weighted MRI (Table 1) [10, 13]. In this study, TIDL was considered grade 3 when CT showed an apparent vertebral endplate fracture (Fig. 2). If both the upper and lower discs were damaged, a more severe TIDL grade was adopted.

**Table 1:** Classification of TIDL

Grade	T2-weighted MRI	Endplate fracture	Characteristic finding
0		None	Intact
1	Hyperintense	None	Edema
2	Hypointense with perifocal hyperintense	None or mild	Disc rupture with intradiscal bleeding
3	Hypointense with perifocal hyperintense	Moderate or severe	Infraction of the disc into vertebral body, annular tears, or infraction without herniation into endplate

*TIDL* Traumatic intervertebral disc lesion

**Fig. 2:**
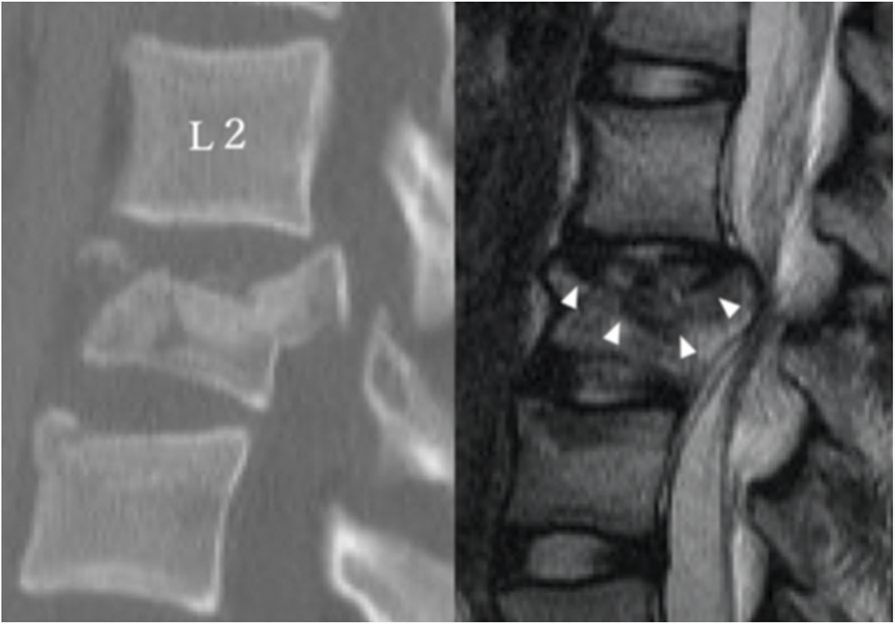
Classification of traumatic intervertebral disc lesion (TIDL). A case of AO type A3 fracture at L3. CT shows a fracture of the cranial endplate and MRI shows infraction of the disc into the vertebral body (white triangles) which means a TIDL grade 3. The caudal disc showed a TIDL grade 2

A case with a depression of 5 mm or more on the sagittal CT slice with the greatest depression was defined to have residual endplate deformity to assess the degree of endplate deformity at follow-up (Fig. 3E).

**Fig. 3:**
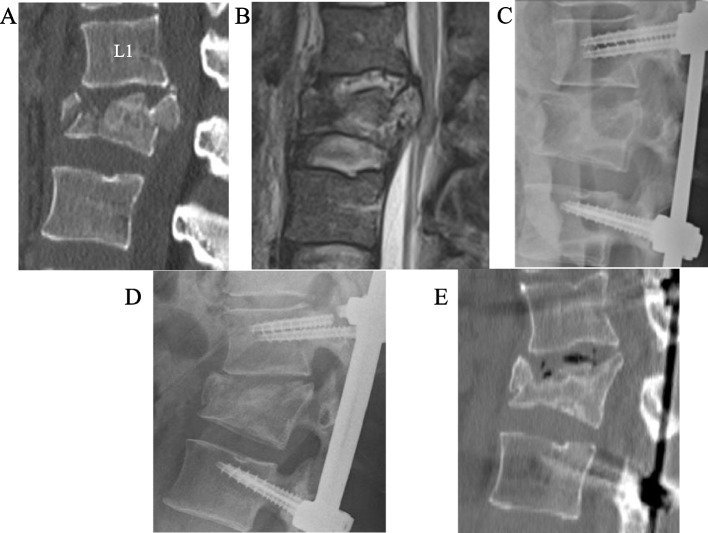
A 39-year-old woman with L2 burst fracture (AO A3). CT (**A**) and MRI (**B**) showed severe damage of the cranial endplate and infraction of the disc into the vertebral body (TIDL grade 3). The fractured vertebra was reduced after surgery (**C**). At follow up, fractured vertebra showed bony union, however disruption of the vertebral endplate and degeneration of intervertebral disc resulted in correction loss and breakage of the pedicle screw (**D, E**). Panel **E** shows residual endplate deformity

### Statistical analysis

The Student’s t-test was performed for continuous normally distributed data, and the Mann-Whitney U test was performed for non-normally distributed data. Categorical data were compared using the chi-square test or Fisher-Freeman-Halton Exact test. Multivariate logistic regression analysis was performed to identify significant risk factors for postoperative correction loss. Statistical analyses were conducted in SPSS for Windows, Version 25 (SPSS Inc., Chicago, Illinois, USA). The level of significance was set at *P* < 0.05.

## Results

### Patient characteristics

In this retrospective study, 48 participants were enrolled, which included 26 men and 22 women. The mean age was 35.0 years (range, 13–63 years) and the mean body mass index (BMI) was 22.0 kg/m^2^ (range, 15.8–28.7 kg/m^2^) (Table 2). The distribution of fractures was as follows: 10 in T12, 17 in L1, 10 in L2, 9 in L3, and 2 in L4. The causes of injury were falls in 33 patients, motor vehicle accidents in 11, and blunt contusions caused by heavy falling objects in 4. Vertebral fractures were classified as follows: 13 patients had type A3, 11 had A4, 11 had B1, and 13 had B2. Implant removal was performed in 38 patients at a mean of 12.8 months after surgery. The mean postoperative follow-up period was 25.7 months (range, 12–98 months).

**Table 2:** Comparison of clinical data between patients with grades 0–2 and grade 3 TIDL

	Total (*n* = 48)	Grades 0, 1, and 2 TIDL (*n* = 28)	Grade 3 TIDL (*n* = 20)	*P*-value
Sex (male/female), n	27/21	16/12	11/9	0.883^a^
Age, years	35.0 (15.5), 30.5, 39.5	34.2 (15.0), 28.3, 40.2	36.1 (16.6), 29.1, 43.2	0.689*
Body mass index, kg/m^2^	22.0 (3.7), 20.9, 23.0	22.6 (3.8), 21.2, 24.0	21.1 (3.6), 19.5, 22.8	0.182**
Follow-up period, months	26.5 (21.9), 20.1, 32.8	26.3 (21.7), 17.9, 34.7	26.7 (22.7), 16.7, 36.7	0.866*
Fracture level (T12, L1/L2–4), n	27/21	19/9	8/12	0.055^a^
AO classification, n				
B1/A3, A4, B2	11/37	11/17	0/20	0.001^b^
LSC score, n				
< 7 / ≥7	28/20	20/8	8/12	0.029^a^
Vertebroplasty, n	17	9	8	0.575^a^
Implant removal, n	38	21	17	0.488^b^
Severe back pain, n	11	1	10	< 0.001^b^

Continuous data are expressed as mean (SD), 95% confident intervals. *P*-values are calculated between the data of patients with TIDL grades 0–2 and grade 3 by Mann-Whitney test*, t-test**, chi-square test^a^, and Fisher’s exact test^b^

*TIDL* traumatic intervertebral disc lesion, *LSC* load sharing classification

### Radiographic outcomes

Overall, SKA and AVBHR significantly improved after surgery, from 11.6° to 3.5° and from 67.2 to 90.0%, respectively. However, the increase in SKA (ΔSKA) and decrease in AVBHR (ΔAVBHR) at follow-up were 10.4° and 9.7%, respectively (Table 3). AVBHR at follow-up was significantly improved than before surgery (*P* < 0.001), whereas there was no significant difference between SKA before surgery and at follow-up (*P* = 0.168). Finally, union of the fractured vertebrae was achieved in 47 patients (98%).

**Table 3:** Comparison of radiological parameters between patients with grades 0–2 and grade 3 TIDL

	Total (*n* = 48)	Grades 0, 1, and 2 TIDL (*n* = 28)	Grade 3 TIDL (*n* = 20)	*P*-values
SKA (degrees)				
Pre-operative	11.6 (11.5), 8.2, 14.9	14.5 (7.0), 10.3, 18.7	7.5 (15.0), 2.5, 12.5	0.037*
At surgery	3.5 (8.6), 1.0, 6.0	6.1 (6.8), 3.0, 9.2	−0.2 (9.6), −3.8, 3.5	0.018*
At follow-up	13.9 (13.1), 10.1, 17.7	13.4 (10.1), 8.4, 18.5	14.6 (16.8), 8.6, 20.5	0.859**
ΔSKA	10.4 (10.9), 7.2, 13.6	7.3 (6.3), 3.4, 11.3	14.7 (14.4), 10.0, 19.4	0.007**
AVBHR (%)				
Pre-operative	67.2 (16.2), 62.5, 71.9	71.5 (14.8), 65.6, 77.4	61.1 (16.6), 54.1, 68.2	0.032*
At surgery	90.0 (11.4), 86.6, 93.3	90.9 (10.4), 86.6, 95.3	88.6 (12.8), 83.4, 93.8	0.507*
At follow-up	80.3, (16.4), 75.5, 85.0	83.4 (16.2), 77.2, 89.5	76.0 (16.1), 68.7, 83.2	0.105**
ΔAVBHR	9.7 (11.0), 6.5, 12.9	7.6 (11.5), 3.5, 11.7	12.7 (9.7), 7.8, 17.5	0.018**
Residual endplate deformity, n		4	17	< 0.001^a^

Data are expressed as mean (SD), 95% confident intervals. *P*-values are calculated between the data of patients with TIDL grades 0–2 and grade 3 by t-test*, Mann-Whitney test** and Fisher-exact test^a^

*TIDL* traumatic intervertebral disc lesion, *SKA s*egmental kyphotic angle, *AVBHR* anterior vertebral body height ratio

### Comparison of clinical data between patients with grades 0–2 and grade 3 TIDL

Twenty patients (42%) showed severe traumatic disc lesions (TIDL grade 3) of the cranial and caudal discs. TIDL grade 3 was significantly more common on the cranial side than on the caudal side (20 vs. 2; *P* < 0.001), and two cases with TIDL grade 3 on the caudal side also showed cranial side lesions (Table 4). Comparing patients with either TIDL grades 0–2 or grade 3, there was no significant difference in gender, age, BMI, fracture level, and application of vertebroplasty (Table 2). However, patients with TIDL grade 3 had a significantly higher LSC score (*P* = 0.029) and a predominance of AO type A3, A4, and B2 fractures compared to AO type B1 fractures (*P* = 0.001).

**Table 4:** Relationship of traumatic lesion grades between the intervertebral discs cranial and caudal to the fractured vertebra

Grade of traumatic intervertebral disc lesion	Caudal disc			
	0	1	2	3
Cranial disc				
0	19	1		
1	1	1	1	
2	2	1	2	
3	14	2	2	2

### Comparison of radiological parameters between patients with grades 0–2 and grade 3 TIDL

Although pre- and postoperative SKA in patients with grades 0–2 TIDL were significantly higher than in patients with grade 3 TIDL (*P* = 0.037 and *P* = 0.018, respectively), ΔSKA during follow-up was significantly greater in patients with grade 3 TIDL (*P* = 0.007; Table 3). Preoperative AVBHR was significantly higher in patients with grades 0–2 TIDL than in patients with grade 3 TIDL (*P* = 0.032) was maintained throughout the course of treatment, and tended to be higher at the final follow-up. ΔAVBHR during follow-up was significantly greater in patients with grade 3 TIDL (P = 0.018). Residual endplate deformity was significantly associated with grade 3 TIDL (*P* < 0.001).

#### Vertebroplasty

The association between vertebroplasty and radiological parameters was analyzed to evaluate the efficacy of vertebroplasty (Table 5). Although AVBHR in patients with vertebroplasty tended to be higher at surgery and follow-up than in patients without vertebroplasty (*P* = 0.064 and 0.082), there was no significant difference in SKA at follow-up (*P* = 0.358). In addition, there was no significant association between vertebroplasty and residual endplate deformity (*P* = 0.732).

**Table 5:** Comparison of radiological parameters between patients with and without vertebroplasty

	Vertebroplasty (+) (*n* = 17)	Vertebroplasty (−) (*n* = 31)	*P*-values
SKA (degrees)			
Pre-operative	12.4 (2.8), 6.7, 18.0	11.1 (2.1), 6.9, 15.3	0.728*
At surgery	3.0 (2.1), −1.2, 7.2	3.8 (1.6), 0.65, 6.9	0.768*
At follow-up	11.4 (3.2), 5.0, 17.8	15.3 (2.4), 10.5, 20.0	0.358*
ΔSKA	8.4 (2.7), 3.1, 13.8	11.5 (2.0), 7.5, 15.5	0.411**
AVBHR (%)			
Pre-operative	62.8 (3.9), 54.9, 70.6	69.6 (2.9), 63.8, 75.4	0.164*
At surgery	94.1 (2.7), 88.6, 99.5	87.7 (2.0), 83.7, 91.7	0.064*
At follow-up	85.8 (3.9), 78.0, 93.7	77.2 (2.9), 71.4, 83.0	0.082*
ΔAVBHR	8.2 (2.7), 2.9, 13.6	10.5 (2.0), 6.5, 14.5	0.502**
Residual endplate deformity, n	8	13	0.732^a^

Data are expressed as mean (SD), 95% confident intervals. *P*-values are calculated between the data of patients with and without vertebroplasty by t-test*, Mann-Whitney test** and chi-square test^a^

*SKA* segmental kyphotic angle, *AVBHR* anterior vertebral body height ratio

### Risk factors for correction loss after surgery

To identify risk factors for correction loss, we compared clinical and radiological parameters between patients with ΔSKA < 10° and ≥ 10° (Table 6). ΔSKA ≥10° was significantly associated with greater ΔAVBHR (*P* < 0.001). ΔSKA ≥10° was significantly associated with older age (*P* = 0.012), smaller preoperative AVBHR (*P* = 0.024), and grade 3 TIDL on the cranial side (*P* = 0.004). Patients with an endplate injury (AO types A3, A4, and B2) were more closely associated with ΔSKA ≥10° than were patients without an endplate injury (AO type B1) (*P* = 0.036) (Fig. 3). However, there was no significant difference in correction loss with regards to gender, fracture level, preoperative SKA, LSC score, and application of posterior decompression and vertebroplasty.

**Table 6:** Comparison between patients with ΔSKA < 10° and ΔSKA ≥10°

	ΔSKA < 10° (*n* = 26)	ΔSKA ≥10° (*n* = 22)	*P*-value
Preoperative SKA (degrees)	11.0 (10.1), 6.9, 15.1	12.2 (13.2), 6.4, 18.1	0.716**
Preoperative AVBHR (%)	72.0 (15.3), 65.8, 78.2	61.5 (15.8), 54.5, 68.5	0.024**
ΔSKA (degrees)	4.6 (3.8), 1.1, 8.2	17.2 (12.7), 13.4, 21.1	< 0.001*
ΔAVBHR (%)	4.2 (4.7), 2.3, 6.1	16.1 (12.7), 10.5, 21.8	< 0.001*
Age, years	30.3 (14.4), 24.5, 36.1	40.5 (15.3), 33.8, 47.3	0.012*
Sex (male/female), n	12/14	15/7	0.125^a^
Fracture level (T12, L1/L2–4), n	15/11	12/10	0.827^a^
AO classification, n			
B1/A3, A4, B2	9/17	2/20	0.036^a^
LSC score, n			
< 7 / ≥7	17/9	11/11	0.281^a^
Vertebroplasty, n	11	6	0.278^a^
Posterior decompression, n	3	4	0.687 ^b^
Implant removal, n	21	17	0.521^b^
Cranial TIDL, n			
Grades 0–2/3	20/6	8/14	0.004^a^
Residual endplate deformity, n	8	13	0.080^b^
Severe back pain, n	2	9	0.006^b^

Continuous data are expressed as mean (SD), 95% confident intervals. *P*-values are calculated by Mann-Whitney test*, t-test**, chi-square test^a^, and Fisher’s exact test^b^

*SKA* segmental kyphotic angle, *AVBHR* anterior vertebral body height ratio, *LSC* load sharing classification, *TIDL* traumatic intervertebral disc lesion

Multivariate logistic regression analysis was used to calculate the adjusted odds ratio (OR) with 95% confidence interval (CI) for risks of ΔSKA ≥10° (Table 7). The presence of cranial grade 3 TIDL and an older age were significantly associated with correction loss.

**Table 7:** Multivariate logistic regression analysis for ΔSKA > 10°

Step	Predictors	*P*-value	Odds ratio	95% CI
1	Cranial TIDL grade 3	0.006	5.83	1.66, 20.56
2	Age	0.026	1.05	1.01, 1.10
	Cranial TIDL grade 3	0.007	6.81	1.71, 27.10

*SKA* segmental kyphosis angle, *CI* confidence interval, *TIDL* traumatic intervertebral disc lesion

### Clinical outcomes

All patients were able to walk with or without assistance during follow-up. No neurological deterioration occurred, and 16 patients who were non-ASIA E preoperatively showed at least one grade of improvement (Fig. 4). The low back pain score was 0, 1, 2, and 3 points in 2, 9, 24, and 13 patients, respectively. Eleven patients (23%) had severe back pain during follow-up. The presence of postoperative severe back pain was significantly associated with grade 3 TIDL at injury (*P* < 0.001) and ΔSKA ≥10° (*P* = 0.006) (Tables 2 and 6).

**Fig. 4:**
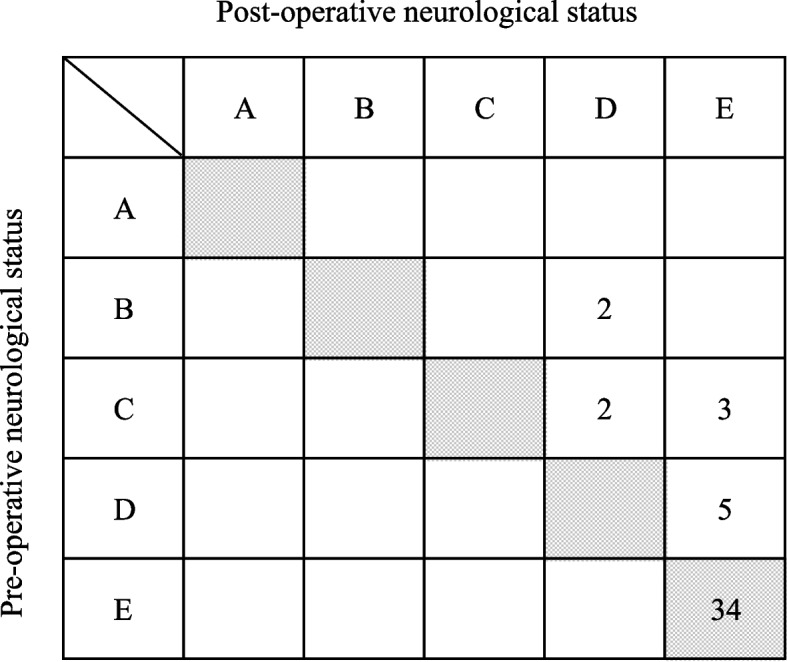
Preoperative and postoperative neurological status using the ASIA impairment scale

### Complications

Eight patients underwent additional procedures. Two patients developed postoperative infections. One patient acquired iatrogenic radiculopathy due to nerve root compression by the leakage of hydroxyapatite blocks for vertebroplasty. Two patients underwent extension of the posterior fusion with instrumentation, and three patients underwent anterior reconstruction due to postoperative delayed kyphotic deformity or nonunion of the fractured vertebra.

## Discussion

The goal of treatment for thoracolumbar burst fractures is to restore the fractured vertebra and prevent future kyphotic deformity. In our series, the AVBHR improved significantly after surgery from 67.2 to 90.0%, which was maintained at 80.3% at the last follow-up. This result indicates that SSPF effectively repairs fractured vertebrae and preserves vertebral body height. Meanwhile, the correction loss of SKA after surgery was 10.4°, consistent with previous reports (ranging from 1° to 13°) [1, 2, 4–9, 20].

Several risk factors associated with correction loss following SSPF have been reported. Aono et al. reported a mean correction loss of 9.1° in SKA for 76 patients. They identified a preoperative SKA greater than 15.4° and canal compromise greater than 52.8% as risk factors [4]. Jang et al. noted that an age greater than 43 years and a preoperative AVBHR less than 54% were risk factors [6]. In this study, the preoperative AVBHR was significantly smaller in patients with ΔSKA ≥10° and older age was a significant risk for correction loss. These results indicate that severely collapsed vertebrae cannot remodel and that the repair capacity of the vertebral body and intervertebral discs is likely to decrease with age.

The previous report indicates that postoperative correction loss occurs at the disc level [4]. Schömig et al. reported that the vacuum disc phenomenon of the adjacent disc often occurred in burst fractures and found a significant correlation between AO A3 fractures and the vacuum disc phenomenon; this may lead to disc degeneration due to nutritional supply disturbances via the vertebral endplate [21]. Other reports have described the importance of reducing the damaged vertebral endplates to prevent progressive kyphotic deformity [22, 23]. Therefore, a preoperative assessment of the endplate and the adjacent disc may be crucial for choosing a treatment strategy and predicting correction loss. Several authors have reported methods for assessing traumatic intervertebral disc injuries [10–13]. Sander et al. classified TIDL according to MRI (Table 1) and reported that 40.7% of their cases were classified as grade 3 at injury [10, 13]. In this series, grade 3 TIDL was observed in 42% of patients, and all patients had cranial injuries. The presence of severe cranial endplate and disc injury (grade 3 TIDL) and an older age were significant risk factors for correction loss. Moreover, cases with AO type A3, A4, and B2 fractures, which accompany the destruction of the vertebral endplate, showed significantly worse correction loss than cases with an AO type B1, which has no endplate injury. Wang et al. reported that endplate fractures were strongly associated with disc degeneration [12]. Therefore, it is presumed that significant endplate damage will also cause considerable damage to the intervertebral discs, resulting in disc degeneration and correction loss at the disc level. Lee et al. showed an association between an intervertebral disc and endplate complex injury and postoperative correction loss [24]. However, their study only included patients younger than 45 and did not assess the correction loss associated with endplate injury and adjacent disc degeneration in older patients.

To our knowledge, this is the first study to investigate the association between the severity of an endplate and adjacent disc injury and correction loss following SPFF at all ages. Therefore, based on our findings, it can be presumed that the damage to either the vertebral endplate, adjacent disc, or both may cause disc degeneration leading to failure of the anterior support mechanism and severe kyphotic deformity. In addition, older patients are at a higher risk because of a reduced ability to repair the discs and endplates.

Excellent surgical outcomes for short-segment instrumentation with vertebroplasty using hydroxyapatite stick have been reported [1]. In this study, vertebroplasty tended to improve and maintain vertebral height, whereas it showed no significant effect in preventing postoperative kyphosis and endplate deformity. The kyphotic deformity was mainly caused by endplate and intervertebral disc injury. Aono et al. also found that postoperative kyphotic change was related to disc level, not to the fractured vertebrae [4]. Vertebroplasty may be effective in preserving vertebral height. However it may not be as effective in preventing kyphosis deformity. If a fractured vertebra has a large cavity after reduction, vertebroplasty may be indicated to preserve vertebral body height.

There is no consensus on whether the residual kyphotic deformity is associated with worse clinical outcomes and back pain [6, 7, 20]. Our study demonstrates that severe postoperative back pain was significantly associated with grade 3 TIDL and ΔSKA ≥10°. Xu et al. noted that postoperative pain was associated with kyphotic deformity greater than 20° but not with the narrowing or degeneration of the intervertebral disc [20]. Similarly, McLain et al. found that a progressive kyphotic deformity of more than 10° was associated with significant postoperative pain [7]. Together, these results demonstrate the association between residual kyphotic deformity and back pain; therefore, it is desirable to prevent excessive correction loss after surgery.

In younger patients or patients without severe TIDL and endplate injury, SSPF is an effective treatment because it can be expected to yield satisfactory results. In contrast, older patients or patients with severe TIDL and endplate injuries are at risk of progressive kyphotic deformity. They can be treated initially with SSPF; however, they require careful observation for correction loss. If severe correction loss occurs, additional surgery should be considered. In older patients with severe TIDL and endplate injury, LSPF or anterior reconstructive surgery may be an effective alternative for initial surgery.

This study had some limitations. First, the retrospective study design may have decreased the level of evidence. Second, the small number of patients evaluated limits the applicability of the study. Despite these limitations, we believe that this study is important. It demonstrated that severe damage to the vertebral endplates and adjacent discs are risk factors for postoperative correction loss and delayed kyphotic deformity.

## Conclusion

Severe intervertebral disc and endplate destruction at injury and an older age are risk factors for correction loss following SSPF for thoracolumbar burst fractures. In younger patients or patients without severe disc and endplate injury, SSPF is an effective treatment. However, in older patients with severe destruction of disc and endplate, LSPF or anterior reconstructive surgery may be a preferred procedure for the initial surgery.

## Data Availability

The datasets generated during and/or analyzed during the current study are available from the corresponding author on reasonable request.

## Abbreviations

SSPF: Short-segment posterior fixation
SKA: Segmental kyphotic angle
AVBHR: Anterior vertebral body height ratio
TIDL: Traumatic intervertebral disc lesion
LSPF: Long-segment posterior fixation
MRI: Magnetic resonance imaging
ASIA: The American Spine Injury Association
JOA: Japanese Orthopaedics Association
CT: Computed tomography
BMI: Body mass index
